# Coronary Microvascular Dysfunction

**DOI:** 10.3390/jcm9092880

**Published:** 2020-09-06

**Authors:** Federico Vancheri, Giovanni Longo, Sergio Vancheri, Michael Henein

**Affiliations:** 1 Department of Internal Medicine, S.Elia Hospital, 93100 Caltanissetta, Italy; 2 Cardiovascular and Interventional Department, S.Elia Hospital, 93100 Caltanissetta, Italy; giova.longo@gmail.com; 3 Radiology Department, I.R.C.C.S. Policlinico San Matteo, 27100 Pavia, Italy; sergiovancheri@gmail.com; 4 Institute of Public Health and Clinical Medicine, Umea University, SE-90187 Umea, Sweden; michael.henein@umu.se; 5 Department of Fluid Mechanics, Brunel University, Middlesex, London UB8 3PH, UK; 6 Molecular and Nuclear Research Institute, St George’s University, London SW17 0RE, UK

**Keywords:** Coronary microcirculation, endothelial dysfunction, microvascular angina, myocardial ischemia without obstructive coronary disease, INOCA, MINOCA

## Abstract

Many patients with chest pain undergoing coronary angiography do not show significant obstructive coronary lesions. A substantial proportion of these patients have abnormalities in the function and structure of coronary microcirculation due to endothelial and smooth muscle cell dysfunction. The coronary microcirculation has a fundamental role in the regulation of coronary blood flow in response to cardiac oxygen requirements. Impairment of this mechanism, defined as coronary microvascular dysfunction (CMD), carries an increased risk of adverse cardiovascular clinical outcomes. Coronary endothelial dysfunction accounts for approximately two-thirds of clinical conditions presenting with symptoms and signs of myocardial ischemia without obstructive coronary disease, termed “ischemia with non-obstructive coronary artery disease” (INOCA) and for a small proportion of “myocardial infarction with non-obstructive coronary artery disease” (MINOCA). More frequently, the clinical presentation of INOCA is microvascular angina due to CMD, while some patients present vasospastic angina due to epicardial spasm, and mixed epicardial and microvascular forms. CMD may be associated with focal and diffuse epicardial coronary atherosclerosis, which may reinforce each other. Both INOCA and MINOCA are more common in females. Clinical classification of CMD includes the association with conditions in which atherosclerosis has limited relevance, with non-obstructive atherosclerosis, and with obstructive atherosclerosis. Several studies already exist which support the evidence that CMD is part of systemic microvascular disease involving multiple organs, such as brain and kidney. Moreover, CMD is strongly associated with the development of heart failure with preserved ejection fraction (HFpEF), diabetes, hypertensive heart disease, and also chronic inflammatory and autoimmune diseases. Since coronary microcirculation is not visible on invasive angiography or computed tomographic coronary angiography (CTCA), the diagnosis of CMD is usually based on functional assessment of microcirculation, which can be performed by both invasive and non-invasive methods, including the assessment of delayed flow of contrast during angiography, measurement of coronary flow reserve (CFR) and index of microvascular resistance (IMR), evaluation of angina induced by intracoronary acetylcholine infusion, and assessment of myocardial perfusion by positron emission tomography (PET) and magnetic resonance (CMR).

## 1. Introduction

Typical angina or atypical symptoms such as exertional dyspnea or episodes of chest pain at rest, are often experienced by patients with coronary artery diseases (CAD) [1,2,3]. Despite signs and symptoms consistent with obstructive atherosclerotic epicardial coronary arteries, about two thirds of women and one third of men with chest pain, and approximately 10% of patients with acute myocardial infarction show no angiographic evidence for significant obstructive coronary lesions [4,5,6,7,8,9,10]. In addition, non-invasive tests (exercise stress test or computed tomographic coronary angiography) have been shown to have limited correlation with the prevalence of obstructive CAD [11]. The rate of patients with myocardial ischemia without epicardial flow-limiting stenosis varies widely because most studies have used different definition of non-obstructive CAD, ranging from <20% lumen stenosis, to ≤50%, or even absence of severe >70% stenosis in any major epicardial coronary artery [10,12,13,14,15]. Additionally, non-obstructive angiograms do not exclude epicardial coronary pathology in the form of diffuse narrowing and incompliant vessels. Moreover, paradoxically increased lumen size may occur during progression of coronary atherosclerosis due to positive arterial remodeling [16].

A substantial proportion (over 50%) of symptomatic patients without flow-limiting coronary lesions have structural or functional abnormalities of the coronary microcirculation leading to impaired vasodilatation, which contributes to myocardial ischemia [17]. Although the coronary microvascular system is one of the main components of coronary circulation, its relevance has been underestimated for a long time, since microvessels are invisible by the current imaging techniques, and their function may be assessed only indirectly.

Coronary microvascular dysfunction (CMD) is defined as the clinical syndrome of angina, electrocardiographic ischemic changes in the absence of obstructive CAD [18]. The pathophysiological basis is impaired microvascular vasodilatation, leading to inadequate increase in blood flow to match myocardial oxygen needs (previously referred to in the literature as “cardiac syndrome X”) [19]. CMD is functionally expressed as reduced coronary flow reserve (CFR), which is the maximum increase in coronary blood flow above the resting value after pharmacological coronary vasodilatation. Reduced CFR due to functional and/or structural abnormalities of the microcirculation has been reported in about 50% of patients with chronic coronary syndromes and up to 20% of those with acute coronary syndromes, in the absence of epicardial coronary flow obstruction [12,20,21,22,23].

Until recently, the prognosis of non-obstructive CAD was thought to be benign, and patients were often inappropriately reassured, without further investigation, despite clinical features requiring coronary angiography. Instead, this condition represents a major cause for myocardial ischemia and is associated with a high risk of major adverse cardiovascular events, including MI, progressive heart failure, stroke, and even sudden death [10,14,24,25,26,27,28]. Moreover, in most patients there is evidence for close interactions between microvascular dysfunction and atherosclerotic epicardial coronary disease [29,30]. CMD is a strong determinant of prognosis even in patients with coronary stenosis of intermediate severity [31].

## 2. Coronary Microvascular Circulation

### 2.1. Distribution of Blood Flow

Coronary microcirculation is represented by pre-arterioles (diameter < 500 µm), arterioles (<200 µm), and capillaries, mostly below the resolution of current angiographic imaging. There is still no available imaging technique that allows for direct morphological visualization of coronary microcirculation. Therefore, its evaluation relies on functional assessment through non-invasive techniques, such as positron emission tomography (PET), or invasive methods [32,33]. Microcirculation has a critical role in the regulation of myocardial blood flow. Since myocardial oxygen extraction is almost maximal at rest (20-fold higher than that of skeletal muscle), increases in oxygen demand can only be met by increases in coronary blood flow. Therefore, to prevent ischemia, an increase in myocardial metabolic activity must be balanced by a proportional increase in coronary blood flow. Because flow resistance is inversely proportional to the fourth power of radius, blood distribution in coronary networks is highly sensitive to arterial diameters. Under resting conditions, the tone of the coronary microcirculation is high. Increased myocardial oxygen demand induces rapid changes in arteriolar diameter, thus allowing the coronary circulation to increase the coronary blood flow.

**Figure 1:**
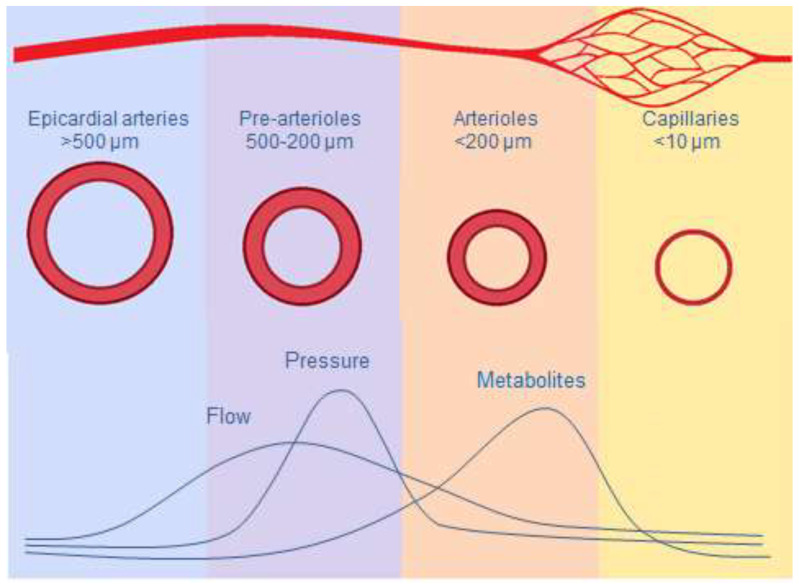
Macro and micro coronary circulation, and mechanisms inducing vasodilatation.

While normally epicardial arteries (5 to 0.5 mm) have a conductance function, exerting minimal resistance to flow, with diameter regulated by shear stress and endothelial function, microcirculation is responsible for most of the vascular resistance of the heart and for distribution of blood according to the oxygen requirements of the myocardium [23]. Different mechanisms allow the microcirculation to regulate myocardial perfusion according to different myocardial metabolic demands (Figure 1).

The pre-arterioles (200–500 µm) are the epicardial component of the microcirculation, responsible for almost 25% of total coronary artery resistance [34]. They maintain pressure of arterioles within a narrow range despite changes in coronary perfusion pressure. Pre-arterioles respond to wall shear stress (WSS) through an endothelium-dependent mechanism, aiming to maintain intraluminal pressure to preserve adequate perfusion pressure in the arterioles [35]. An increase in flow rate induces vasodilatation, whereas a reduction induces vasoconstriction.

Arterioles (40–200 µm) represent the intramyocardial component of the microcirculation, accounting for more than 50% of the total coronary arterial resistance. Their function matches myocardial blood supply and oxygen consumption. Although there is some overlap between the mechanisms that regulate blood flow in these vessels, the diameter of large arterioles (100–200 µm) is mainly regulated by endothelium-dependent flow dilatation, mediated by WSS through endothelial surface receptors. In medium-sized arterioles (40–100 µm) the main mechanism involved in regulating microvascular circulation is independent of endothelial function and based on intraluminal pressure changes detected by smooth muscle cell stretch receptors (myogenic control). The artery constricts when intraluminal pressure increases and dilates when pressure decreases [36]. The tone of the smallest arterioles (<40 µm) is modulated by substances produced by the myocardial metabolic activity. As a result of this fine-tuned mechanism, increased myocardial metabolic activity promotes vasodilatation of the smaller arterioles, leading to intraluminal pressure reduction in medium-sized arterioles due to myogenic dilatation. This in turn increases flow upstream in the large arterioles through endothelium-dependent vasodilatation. Through these mechanisms, micro circulation regulates myocardial perfusion, both at rest and at different levels of myocardial metabolic demands, allowing up to a five-fold increase in coronary blood flow [37,38,39].

### 2.2. Heterogeneity of Coronary Microcirculation

Since myocardial metabolism is aerobic with high baseline oxygen extraction, there is almost a linear relationship between oxygen demand and coronary blood flow. However, the local perfusion of the myocardium is highly heterogeneous, with high and low oxygenated areas, even in normal heart [40,41,42]. Indeed, experimental studies have shown that in physiological conditions, both blood flow and contractile function are unevenly distributed throughout the left ventricle (LV). Midwall segment and circumferential shortening are higher at the apex and in the anterior LV wall compared to the base and the posterior wall, respectively [43]. There is also evidence for transmural heterogeneity due to reduced myogenic response in the subendocardium compared with the subepicardium. This may account for the greater vulnerability of subendocardium to ischemic injury. Indeed, in experimental settings, myocardial hypoperfusion due to coronary obstruction causes more pronounced reduction in subendocardial blood flow and contractility compared to subepicardial regions [43]. Heterogeneity is also the result of adaptive processes of microcirculation in response to functional demands. These include fast changes in arteriolar diameter due to changes in smooth muscle tone and slower long-term structural microvascular remodeling with the addition or removal of vessels by angiogenesis or vascular pruning [44]. Microvascular dysfunction causes the development of functional shunting, increasing the heterogeneity of flow and oxygen and local myocardial supply-to-demand mismatch. Hence, the regions of abnormal perfusion have a patchy distribution, thereby explaining the normal findings of diagnostic imaging that do not provide the resolution to evaluate small perfusion segments [45].

### 2.3. Collaterals

In patients with obstructive coronary artery disease (CAD), the presence of anastomoses between epicardial coronary arteries may serve as a bypass to provide alternative blood supply to myocardial regions distal to the obstruction, thereby preventing myocardial ischemia or reducing its extension [44,46]. Collaterals result from pre-existing small arterial interconnections (arteriogenesis), which remodel into functional arteries in response to raised WSS on the collateral vessel endothelium due to enhanced flow resulting from an increased pressure gradient between pre- and post-obstructive vessels [47,48,49]. Well-developed collaterals capable of preventing myocardial ischemia during brief coronary occlusion have been demonstrated in only one third of individuals with angina and angiographically normal coronary arteries [50]. However, collaterals can provide less than 40% of maximal normal blood flow [51]. An alternative, less frequent mechanism is the growth of new capillaries by hypoxia-induced sprouting from existing vessels (angiogenesis) [48,52]. Although collateral circulation can supply only part of the metabolic needs of the ischemic myocardium, a high degree of collateralization has been shown to improve survival in patients with stable or acute CAD [53,54].

## 3. Pathophysiology of CMD

In contrast to coronary epicardial atherosclerosis, CMD does not develop atheroma. However, coronary microcirculation can develop structural and functional atherosclerotic changes, particularly in patients with cardiovascular (CV) risk factors. CMD is expressed as either the inability of the coronary arteries to dilate appropriately to meet myocardial oxygen demand (vasodilator abnormality) and/or as the abrupt reduction in coronary blood flow (coronary microvascular spasm). The underlying mechanism for coronary vasomotor dysfunction may be endothelium-dependent or endothelium-independent. Endothelium-dependent dysfunction is a consequence of an imbalance between endothelium-derived relaxing factors, such as nitric oxide (NO), and endothelium-derived constrictors, such as endothelin. Endothelium-independent function is based on myocyte tone. Moreover, CMD can occur in the absence and/or in the presence of obstructive epicardial coronary artery disease [55]. The myocardial consequences of coronary blood flow abnormalities related to CMD are different from those caused by epicardial flow-limiting stenosis. In the latter, the impairment in myocardial perfusion is homogeneously distributed in the region perfused by the stenosed artery, resulting in segmental impairment of wall contraction. In contrast, the myocardial ischemia in CMD may not involve all microvessels originating from an epicardial artery but may have a patchy distribution. This explains why these patients may have symptoms without myocardial wall contraction abnormalities [56].

### 3.1. Endothelial Dysfunction

Despite the fact that endothelial structure and function may vary considerably among different vascular regions, they have a common feature in secreting substances that regulate vascular tone and permeability, thus contributing to vascular protection [57]. The endothelium has a crucial role in CV homeostasis, in particular in the myocardial capillaries where endothelial cells are directly in contact with adjacent cardiomyocytes [58]. Normally, under physiological stimuli such as exercise, the vascular endothelial cells modulate an appropriate dilatation of coronary arteries by locally releasing vasodilator substances, in particular NO [59,60,61]. NO also protects the integrity of the endothelium through its anti-inflammatory properties by inhibiting fibrosis, platelet aggregation, and apoptosis and by promoting angiogenesis.

CMD is initiated by the low-grade inflammatory response to CV risk factors, similar to those of obstructive CAD, although they have been shown to account for <20% of variability in microvascular function, leaving a large proportion unexplained [62,63,64]. The inflammatory response reduces NO bioavailability, which is the link between atherosclerosis and microvascular dysfunction, leading to impairment of both endothelium-dependent and endothelium-independent coronary microvascular vasomotor function, thus increasing the risk of myocardial ischemia. Functional changes induced by endothelial dysfunction may be simultaneously present in coronary microcirculation and in the epicardial arteries, as well as peripheral vascular sites, thus representing a systemic disorder [65,66]. Inflammation-induced endothelial dysfunction is thought to be mediated by elevated C-reactive protein (CRP) levels [62,67,68,69]. However, it is still controversial whether high CRP concentration is cause or effect of atherosclerosis, or confounding factor [70,71,72].

The inflammatory response of endothelial cells is strongly influenced by WSS, which is the tangential component of the mechanical friction exerted by the flowing blood on the vascular endothelial surface [73,74]. Specific biomechanical receptors in the glycocalyx, a surface proteoglycan layer, translate WSS into biochemical signals that modulate the vascular tone, platelet activity, leukocyte adhesion, and endothelial permeability [75]. While physiological WSS promotes the maintenance of anti-inflammatory properties of endothelial barrier, low WSS induces inflammation, and atherogenesis through decreased endothelial production of NO [76,77]. This results in impairment of dilator function and blunted coronary blood flow augmentation in response to myocardial metabolic demands. Impaired endothelial function may also release vasoconstrictor substances (endothelin, prostaglandin, and thromboxane), thus shifting the vasomotor response to physiological stimuli from dilatation to constriction, reducing the blood flow [78]. Vasomotor abnormalities in patients with CMD include coronary spasm, which affects large and small coronary arteries. Following intracoronary infusion of acetylcholine, about 50% of patients with exertional angina and no evidence of obstructive CAD develop coronary microvascular spasm, defined as angina and ischemic ECG changes without changes in epicardial coronary artery diameter [15].

The decreased production of NO by impaired endothelial cells also increases collagen deposition, reduces angiogenesis and collateral development, and promotes the conversion of endothelial cells into mesenchymal cells, leading to microvascular rarefaction defined as loss of perfused microvessels, fibrosis, and hypertrophy [79,80]. Moreover, while under normal conditions endothelial cells produce anti-proliferative and antithrombotic mediators, inflammation promotes a switch in endothelial function from a quiescent to an activated state, involving platelet activation, lipid oxidation, and leukocyte adhesion and migration which, in turn, accelerate inflammation [81,82]. Microvascular inflammation and reduced NO availability directly promote proliferation of fibroblasts, which increase the production of fibronectin and collagen leading to cardiac remodeling following myocardial infarction, pressure overload, and myocarditis [83,84,85]. Additionally, proliferation of fibroblast and increased content of extracellular matrix proteins increase the distance of oxygen diffusion between capillaries and myocytes, exposing the myocardium to the risk of hypoxia under the condition of reduced blood flow.

Microvascular functional abnormalities, characterized by persistent changes in microvascular tone, are also associated with the development of structural abnormalities, which include luminal narrowing, due to inward remodeling of intramyocardial arterioles, rarefaction of microvessels, and microembolization after atherosclerotic plaque rupture or during coronary intervention. Remodeling is mainly caused by medial wall thickening because of proliferated smooth muscle cells, surrounded by perivascular fibrosis [86,87]. Both arteriolar remodeling and decrease in capillary density (estimated as capillary number per unit area) contribute to increased microvascular resistance and reduced CFR [88,89,90,91]. Coronary microvascular microembolization by debris from epicardial atherosclerotic plaques together with thrombogenic and inflammatory substances may develop acutely following plaque rupture or erosion [92,93]. This may occur spontaneously or may be induced during percutaneous coronary intervention after myocardial infarction. Release of debris and thrombogenic substances from atherosclerotic epicardial lesions may also be chronic and asymptomatic, leading to progressive microvascular dysfunction.

### 3.2. Autonomic Nervous System

Sympathetic and parasympathetic nerves can modulate arterial tone directly acting on vascular smooth muscle cells or stimulating the release of NO from the endothelium [36]. The physiological role of coronary blood flow control by the vagus nerve is uncertain. However, under controlled conditions, the intracoronary infusion of acetylcholine results in dilatation of resistance vessels and a three to four-fold increase in blood flow. In contrast, in patients with coronary atherosclerosis, the response is attenuated or results in epicardial or microvascular coronary spam [15]. Such effects of acetylcholine on the coronary endothelium are used to test the endothelium-dependent coronary function.

At rest, the sympathetic control of coronary vasomotor tone is negligible and depends on the balance between β-adrenergic mediated arterial dilatation, which is prevalent, and α-adrenergic mediated vasoconstriction that has only a limited effect [36]. During exercise, coronary tone is modulated by sympathetic activation through release of norepinephrine by sympathetic nerves of coronary circulation, as well as by circulating norepinephrine and epinephrine. β-adrenergic activation, involving mainly β_2_-adrenoceptors, contributes to coronary vasodilatation to compensate for the increased myocardial oxygen consumption, accounting for approximately 25% of exercise hyperemia [94]. However, when the coronary circulation is impaired by atherosclerotic endothelial dysfunction, the α_1_-adrenergic mediated vasoconstriction becomes more intense, reduces blood flow, and may lead to myocardial ischemia [95,96].

## 4. Clinical Presentation

Conditions presented as symptoms and signs of myocardial ischemia without significant coronary artery stenosis (<50% diameter stenosis) are termed “ischemia with non-obstructive coronary artery disease” (INOCA) [20,27,97,98]. Endothelial dysfunction accounts for approximately two-thirds of INOCA [99]. Among these patients, the clinical feature of microvascular angina (MVA), defined as the clinical manifestation of myocardial ischemia due to CMD in the absence of flow-limiting CAD [18,100], is present in 52%, vasospastic angina (VSA) defined as a manifestation of epicardial spasm in 17%, and mixed forms in 20% [101,102].

**Figure 2:**
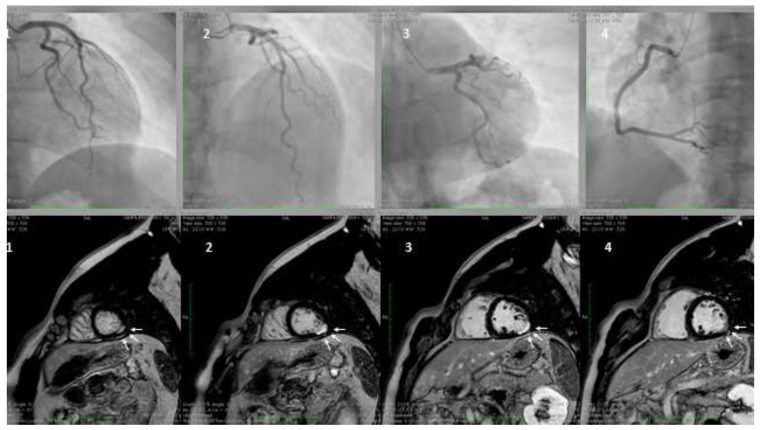
A hypertensive, smoker, young male patient was admitted for inferior ST-elevation myocardial infarction (STEMI). Coronary angiography was performed with no coronary lesions on the left (panel A, 1,2, and 3) and right (panel B, 4) coronary artery. Subsequentially, cardiac magnetic resonance (CMR) was performed. Delayed enhancement sequences, from apex to base, show sub-endocardial lesions (white arrows, image 1 and 4) with transmural extension (white arrows 2 and 3), confirming the ischemic lesions of the inferior-lateral wall.

Myocardial infarction with non-obstructive coronary artery disease (≤50% diameter stenosis in a major epicardial artery) is termed MINOCA (Figure 2). This is a heterogeneous group of conditions including epicardial and microvascular causes of myocardial ischemia, such as plaque disruption, epicardial coronary spasm, spontaneous coronary dissection, microvascular spasm, and coronary distal embolization [2,4,103,104,105,106,107]. The features of Takotsubo cardiomyopathy and myocarditis, initially included in the definition, were subsequently excluded [2]. The prevalence of MINOCA is approximately 10% of all type 1 myocardial infarction (MI), more common in women and in patients presenting with Non-ST-elevation myocardial infarction (NSTEMI) [108,109]. CMD, expressed by coronary microvascular spasm, accounts for about 20% of MINOCA patients [4,108,110]. A seasonal pattern of occurrence with a peak in summer and autumn has been observed in patients with MINOCA [111]. In contrast, it is still uncertain whether MI due to CAD is equally distributed across seasons or more frequent in winter, in association with respiratory tract infections. These differences may be related to different pathophysiologic triggers leading to MI, e.g., microvascular spasm in patients with MINOCA or atherosclerotic plaque rupture in MI due to CAD.

CMD may occur in asymptomatic individuals and may be diagnosed incidentally. Symptoms of myocardial ischemia are indistinguishable from those caused by epicardial stenosis. One to two-thirds of patients present typical exertional angina, more common in postmenopausal women than in men [112]. Atypical symptoms, including retrosternal chest pain at rest or angina-equivalent, such as dyspnea on exertion, are also common. Effort-induced symptoms tend to occur in the post exercise recovery period, due to persistence of imbalance between metabolic demand and oxygen delivery. Nitrates are less effective in relieving symptoms because their vasodilator effect is more pronounced in the epicardial arteries compared to microvascular circulation. Standardized criteria for clinical diagnosis of MVA have been developed by the “Coronary Vasomotion Disorders International Study Group” (COVADIS), based on symptoms, absence of obstructive CAD, evidence of myocardial ischemia, and evidence of microvascular dysfunction (Table 1) [18].

### 4.1. Gender Differences in CMD

Several studies have shown that females have a lesser extent of obstructive CAD compared with males [25,109]. In contrast, they have higher prevalence of angina without obstructive CAD [19,113,114]. In the presence of non-obstructive CAD, myocardial ischemia may be due to microvascular endothelial dysfunction, epicardial and microvascular spasm, or conduit artery stiffening. Moreover, in patients without angiographically obstructive CAD, intracoronary imaging studies using IVUS (intravascular ultrasound), which allows direct cross-sectional visualization of the arterial wall, have shown high prevalence of positive remodeling and preserved lumen size [29,30]. This may explain the absence of flow-limiting lesions which can only outline contrast-filled coronary lumen. These features are more frequent in women and are associated with increased risk of CV events [115,116]. Additionally, women have higher prevalence of MINOCA—10.5% compared to 3.4% for men [4,117].

Symptomatic women are generally older than men and often have greater number and severity of CV risk factors. Moreover, most patients are in the peri- or post-menopausal stage, suggesting that reduced levels of estrogen have a role in the development of CMD [118]. Angina in the absence of obstructive CAD is also psychologically relevant, as recognition of the ischemic heart disease may be delayed. This may induce anxiety in the patient because there is no clear diagnosis, thus reducing the patient’s quality of life.

### 4.2. Clinical Classification

CMD includes a wide spectrum of conditions variously associated with atherosclerosis. Accordingly, the traditional CMD classification takes into account the clinical and pathophysiological setting in which it may occur [23,119]. This classification has been recently updated in relation to the severity of CAD [20] (Table 2).

In addition, because CMD may be associated with clinical conditions in which atherosclerosis is minimal or absent or associated with obstructive or non-obstructive coronary atherosclerosis, a simplified classification has been proposed, including three main CMD phenotypes: without atherosclerosis, with non-obstructive atherosclerosis, and with obstructive atherosclerosis [78].

## 5. CMD without Atherosclerosis

CMD is frequently associated with clinical conditions in which atherosclerosis has limited relevance, such as arterial hypertension, aortic stenosis, and non-ischemic cardiomyopathies including idiopathic, hypertrophic, infiltrative, and stress cardiomyopathies [120]. In these conditions, it is unknown whether CMD is a cause or consequence of the underlying myocardial disease. However, CMD is thought responsible for subendocardial ischemia, diffuse interstitial fibrosis, and impairment of LV function [120,121,122,123,124,125,126].

## 6. CMD with Non-Obstructive Atherosclerosis

CMD is part of systemic microvascular diseases, which affects multiple organs. Several cardiac and systemic conditions, such as heart failure with preserved ejection fraction (HFpEF), brain small-vessel disease, diabetes, hypertensive heart disease, chronic kidney disease, and chronic inflammatory and autoimmune diseases, can develop myocardial ischemia due to CMD without significant epicardial coronary obstruction. These conditions are quite prevalent and associated with substantial risk of acute CV events.

### 6.1. CMD in the Development of HFpEF

Several clinical studies indicate that CMD has a central role in the pathogenesis of HfpEF [127,128,129,130]. Indeed, it has been proposed that microvascular angina and HFpEF are the extreme clinical manifestations of the same spectrum of disease [131]. Cardiomyocytes are in close proximity—less than 3 µm—to endothelial cells. Such an anatomical arrangement allows for adequate blood supply and bidirectional influences through the release of vasoactive substances, including NO, natriuretic peptides, cytokines, and endothelin-1.

Microvascular inflammation and dysfunction is triggered by low-grade systemic inflammation induced by conventional CV risk factors, including hypertension, diabetes, obesity, dyslipidemia, and also other conditions associated with chronic inflammatory and autoimmune rheumatic diseases [132,133]. It has been observed that CRP, a biomarker of systemic inflammation produced in response to interleukin-6 by inflammatory cells, has both diagnostic and prognostic value in predicting the risk of developing HFpEF and subsequent CV events. However, the results of the studies are conflicting [134].

Inflammation-induced endothelial dysfunction reduces microvascular NO bioavailability and the cardiomyocyte content of cyclic guanosine monophosphate (cGMP), protein kinase G (PKG), and transforming growth factor (TGF)-β, which are involved in several cardiac physiological processes, including the regulation of cardiac hypertrophy and stiffness [135,136,137,138]. Since in normal conditions NO has direct anti-fibrotic effects, its reduction promotes fibrosis. Furthermore, NO reduction impairs cGMP and TGF-β functions, and favors conversion of endothelial cells into mesenchymal cells that can give rise to fibroblasts [80,138,139]. Overall, these changes promote hypertrophy and fibrosis, thus contributing to left ventricular (LV) diastolic dysfunction.

Both cardiac fibrosis and cardiomyocyte hypertrophy are responsible for subendocardial ischemia as reflected in impaired left ventricular (LV) longitudinal systolic abnormalities, diastolic dysfunction, and remodeling, leading to HFpEF, LV stiffness, and increased filling pressure [128,140]. These abnormalities, in turn, may trigger further subendocardial ischemia and endothelial dysfunction [141].

Inflammation accounts also for the differences in myocardial remodeling between HFpEF and HF with reduced ejection fraction (HFrEF). While HFpEF is associated with concentric hypertrophy, adverse remodeling in HFrEF is usually due to progressive loss of cardiomyocytes as a result of ischemia, with replacement of dead cells by collagen, forming patchy areas of replacement fibrosis, leading to LV dilatation and maladaptive remodeling [85,88,127,142,143].

Based on the differences in pathophysiology between HFpEF and HFrEF, the biomarkers of inflammation, including high-sensitivity CRP and interleukin-6, have been found higher in HFpEF than in HFrEF, whereas markers of cardiomyocyte injury, such as brain natriuretic peptides (BNPs) and high-sensitivity troponin T, are higher in HFrEF than in HfpEF [144].

### 6.2. Microvascular Disease of the Brain

Most patients with CMD also have abnormalities of perfusion in the brain microcirculation [145,146]. The heart and brain share similar vascular anatomy. Large conduit arteries are distributed on the surface of both organs, while vascular tone regulation is carried out by perforating arteries. Cerebral small vessel disease (SVD) refers to a clinical syndrome involving cognitive, neuroimaging, and pathological findings arising from disease of small perforating cerebral arteries [145]. It is associated with ageing and conventional CV risk factors, although these can explain only a small proportion of the variance [147]. SVD may present with focal neurological symptoms in the form of lacunar stroke, defined as small infarct (<15 mm) of the cerebral white matter or basal ganglia, which account for one-quarter of ischemic strokes. It may also present with diffuse neurological symptoms and progressive cognitive decline, gait disturbance, and frank dementia, referred to as leukoaraiosis on computer tomography or magnetic resonance imaging [148,149,150,151,152]. Lacunar strokes contribute to up to 45% of cases of dementia [153,154]. Only a small proportion of lacunar strokes arise from atherothrombosis and embolism, while most of them seem to result from the diffuse underlying dysfunction of cerebral small vessels [155].

The prevalent histological abnormality in lacunar strokes is a diffuse disease of the small arterioles (40–200 µm diameter), termed arteriolosclerosis, consisting of concentric hyaline thickening of small penetrating arteries (lipohyalinosis), associated with fibrosis, reduced smooth muscle cells, or fibrinoid necrosis. The diseased vessels also include infiltration of inflammatory cells in the arterial wall and perivascular tissue [145]. These changes are thought to be due to arteriolar and capillary endothelium dysfunction [156]. Cerebral endothelial cells modulate vascular tone and blood flow, exert anti-inflammatory effects, protect against thrombosis and fibrosis, and promote clearance of amyloid peptides [157]. In addition, the cerebral microvascular endothelium has a peculiar function contributing to blood–brain barrier (BBB) structure, along with perivascular space, pericytes, and astrocyte end-feet [150,158]. The interplay of all these components creates an interface between blood and brain to maintain neurovascular homeostasis.

Systemic or vascular inflammation increases the permeability of BBB, resulting in leakage of plasma components and migration of inflammatory cells into the vessel wall [159,160,161,162]. Loss of normal endothelial function could, therefore, result in small vessel structural and functional changes, together with perivascular edema, which may lead to lacunar stroke and leukoaraiosis [155,163,164]. Moreover, BBB leakage promotes extracellular accumulation of amyloid-β, which has a pathogenetic role in Alzheimer’s disease [165]. This accumulation, in turn, impairs endothelial structure and function. BBB impairment may be a link between endothelial dysfunction and neurodegenerative diseases.

The vessel lumen restriction in SVD causes chronic hypoperfusion of the white matter, resulting in ischemic lesions and death of oligodendrocytes with degeneration of myelinated fibers. Persistence of BBB dysfunction worsens microvessel damage, promoting secondary inflammation and further impairment of vascular tone, luminal narrowing, and tissue ischemia [153].

Microcirculation also has an important role in the brain reperfusion injury shortly after ischemia, referred to as the no-reflow phenomenon, similar to that occurring in the heart [166,167]. However, there are some differences due to the composite cellular structure of BBB. Recanalization of the occluded artery does not necessarily lead to reperfusion, which is the condition more strongly associated with clinical outcomes.

Experimentally, cellular changes associated with no reflow may be observed after 5–10 min of ischemia. Within the first minutes of flow cessation, there are significant alterations in microvascular permeability [168]. Recanalization of the occluded artery and restoration of flow is followed by a brief period of hyperperfusion. However, over the next 30 min hypoperfusion occurs, and in the subsequent 6 h oxygen levels decrease below those observed during initial ischemic occlusion [169]. Endothelial cells and astrocyte end-feet undergo swelling, resulting in decreased luminal size, which impairs blood flow. During reperfusion, the return of oxygenated blood to the ischemic area activates coagulation factors and inflammatory cells, obstructing the microvessel lumen, leading to further increase in BBB permeability [158]. In addition to blood elements, pericytes are critical for stabilization of the capillary wall and have been shown to have an essential role in reperfusion injury [170]. These cells contain contractile proteins and closely interact with the vascular endothelium, regulating cerebral microvascular blood flow, similar to smooth muscle cells in large arteries. During ischemia, pericytes contract and are no longer able to relax despite recanalization of the occluded artery, thus impeding microcirculation and oxygen supply to the brain [171,172]. In this experimental model, the level of inflammation and neuronal damage has been shown to correlate more with no reflow events than with acute occlusion damage. The extent of brain damage depends also on the duration of blood flow interruption before reperfusion [173]. After long-lasting focal ischemia, the microcirculatory changes are irreversible. However, after brief focal ischemia, the no reflow changes are potentially reversible, defined as “incomplete microcirculatory reperfusion”, localized in penumbra areas [174].

### 6.3. CMD in Diabetes

Impaired coronary microvascular function has been demonstrated in type 2 (T2DM) and type 1 diabetes (T1DM), even without hypertension or dyslipidemia [175,176,177]. The relationship with T2DM is bidirectional [55]. Chronic hyperglycemia is associated with significantly reduced endothelial-dependent and endothelial-independent coronary vasodilatation [119]. Furthermore, insulin resistance and hyperinsulinemia contribute to impair the microvascular endothelium-dependent coronary and skeletal vasodilatation in prediabetes through increased oxidative stress, inflammation, and dyslipidemia [178]. CMD, in turn, contributes to the progression of prediabetes to T2DM by reducing the delivery of insulin and glucose to skeletal muscles. When stratified by the severity of metabolic impairment, patients with diabetes or metabolic syndrome show a stepwise increase in microvascular dysfunction and risk of coronary events [179]. Overweight and obesity, commonly associated with T2DM, are characterized by epicardial and perivascular accumulation of adipose tissue, which promotes inflammation and progression of coronary atherosclerosis [180,181].

### 6.4. CMD in Hypertensive Heart Disease

Hypertension entails hemodynamic and neurohumoral effects that trigger adaptive LV remodeling. In addition, the hemodynamic load of hypertension may induce rarefaction of coronary microcirculation leading to impairment of myocardial perfusion [182]. The impairment of CFR in hypertensive patients is independent of the presence and degree of LV hypertrophy (LVH), suggesting that CMD is the result of functional alterations of endothelial and smooth muscle cells, leading to vascular remodeling and increased microvascular resistance [183]. However, the progression from hypertension to hypertensive heart disease and heart failure cannot be fully explained by the myocardial response to elevated blood pressure [184,185]. The development of concentric or eccentric LV hypertrophy (LVH) depends on the etiology of hypertension and interplay of pressure and volume overload. Compared to patients with essential hypertension, those with renovascular hypertension have greater prevalence of LV concentric hypertrophy and diastolic dysfunction. In addition to microvascular rarefaction, LV hypertrophy includes interstitial fibrosis and myocyte hypertrophy, resulting in increased intercapillary distance and decreased oxygen delivery. Furthermore, the progression from hypertension to heart failure is unpredictable, occurring in patients with maladaptive remodeling but also in those with compensatory remodeling and even in patients with normal LV function and geometry [186]. In addition, the mechanisms involved in the transition from hypertensive heart disease to heart failure are uncertain. While a small proportion of patients with LV hypertrophy undergo myocardial infarction developing LV dilatation and heart failure, most patients progress directly to heart failure [187]. Abnormalities in microvascular function, structure, and perfusion may be responsible for the transition from hypertensive heart disease to LV dysfunction and heart failure in patients without epicardial CAD [188,189]. Microvascular rarefaction, perivascular fibrosis, and medial thickening of arterioles with reduced luminal areas have been observed in patients with hypertension without obstructive CAD [190,191]. These changes increase microvascular resistance, reducing blood flow. Furthermore, the endothelial dysfunction impairs flow-mediated vasodilatation of arterioles, leading to chronic subendocardial ischemia and impaired myocardial mechanical function [130,192].

### 6.5. CMD in Hypertrophic Cardiomyopathy (HCM)

Symptoms and signs of myocardial ischemia, despite non obstructive coronary angiograms, are frequent findings in patients with hypertrophic cardiomyopathy (HCM). In these patients, CMD is associated with myocardial ischemia and may contribute to some complications, including systolic and diastolic dysfunction, progressive LV remodeling, and ventricular arrhythmias [23]. Both hemodynamic factors and coronary structural abnormalities induce CMD and myocardial ischemia in patients with HCM. In the normal heart, coronary flow is mainly determined by the forward compression wave generated by ventricular contraction, and the backward expansion wave resulting from the decompression of the microcirculation as the ventricle relaxes [193]. In patients with HCM, compression of the intramyocardial arterioles during ventricular systole greatly reduces coronary flow. In the presence of LV outflow tract obstruction, blood flow is further decreased and can even reverse in the epicardial arteries [194]. Additionally, impaired ventricular relaxation reduces the coronary flow during diastole. However, coronary flow is impaired not only in the severely hypertrophied septum but also in the less hypertrophied LV free wall [132]. Indeed, in addition to hemodynamic impairment, HCM involves marked structural abnormalities of coronary microcirculation, including medial hypertrophy, intimal hyperplasia, interstitial fibrosis, and myocyte disarray, resulting in reduced coronary luminal area and lower capillary density [55,195,196]. These microvascular structural changes are strongly associated with long-term adverse LV remodeling and dysfunction [197].

### 6.6. CMD in Aortic Valve Stenosis

Angina is reported in approximately 40% of patients with aortic valve stenosis in the absence of coronary flow-limiting lesions at angiography [39]. Under these circumstances, myocardial ischemia is caused by CMD. Patients with aortic stenosis develop adaptive LV hypertrophy (LVH) to reduce LV wall stress. The development of LVH and the pressure drop across the aortic valve affect the coronary circulation, reducing the CFR. Several mechanisms are involved, including reduced diastolic coronary filling time, increased LV filling pressure, and intramyocardial pressure, resulting in subendocardial perfusion impairment and reduced systolic acceleration of coronary flow, resulting in low coronary perfusion pressure compared with intra-ventricular pressure, increased intramyocardial systolic pressure, and delayed myocardial relaxation at the end of systole, further reducing the time of coronary filling and reducing capillary density [39,198]. Additionally, the study of myocardial blood flow (MBF), using positron emission tomography, has shown that MBF increases proportionally with LV mass, despite reduced capillary density [199]. Therefore it is conceivable that the increase in resting MBF is due to metabolic vasodilatation in response to the increased oxygen demand of LVH. This mechanism makes microcirculation unable to further increase blood flow, contributing to limit CFR. However, the reduction of CFR is more closely related to the severity of aortic stenosis, hemodynamic load, and reduced diastolic perfusion time, than to the increase in LV mass [55,119,199,200]. Unlike HCM, coronary microcirculation in aortic stenosis does not develop structural changes like medial hypertrophy or perivascular fibrosis [201]. In contrast, fibroblasts, myofibroblasts, and the external matrix are increased [39].

### 6.7. CMD in Chronic Kidney Disease (CKD)

Chronic kidney disease (CKD) and CMD are strongly associated and enhance each other [55,202]. CKD induces hypertension through neuro-humoral pathways and contributes to LV hypertrophy, decreased cardiac function, diastolic dysfunction, and increased risk of adverse CV events, referred to as Type 4 cardiorenal syndrome [203]. In turn, traditional CV risk factors, especially hypertension and diabetes, have been considered the main causes of the development of CKD. However, impaired kidney function may promote CV disease independent of traditional CV risk factors [204]. There is evidence that minor renal abnormalities such as slightly reduced estimated glomerular filtration rate (eGFR) or microalbuminuria, even within the normal range, are associated with increased risk of CV events, independently of risk factors [205]. Clinical studies have shown that in patients without obstructive epicardial disease, CMD is the link between impaired renal function, myocardial dysfunction, and CV events [206], and the severity of its impairment is associated with the CKD stage [207,208]. Endothelial dysfunction is the pathophysiological mechanism involved in the association between CMD and CKD. The kidney has an extensive vascular supply with the largest total endothelial surface area in the body. In patients with CV risk factors, especially hypertension and diabetes, endothelial dysfunction induces vascular rarefaction in glomerular capillaries, causing tissue hypoxia and kidney damage [209]. In patients with progressive CKD, uremia-specific risk factors, activation of the renin–angiotensin system, oxidative stress, and low-grade inflammation promote coronary microvascular rarefaction and myocardial fibrosis [210,211,212,213].

### 6.8. CMD in Chronic Inflammatory and Autoimmune Diseases

There is ample evidence that patients with chronic inflammatory and autoimmune disorders, such as rheumatoid arthritis, systemic lupus erythematosus, systemic sclerosis, ankylosing spondylitis, inflammatory bowel disease, psoriasis, and periodontitis, have increased risk of CV diseases [214,215,216,217,218,219]. Part of this risk is mediated by common CV risk factors but mostly depends on inflammation as a risk factor for CV disease [220,221]. Endothelial dysfunction is the pathophysiological link between inflammatory diseases and both macrovascular and microvascular impairment [55,222,223]. Indeed, significant reduction of CFR has been observed in patients with inflammatory disease, even in the early stages of the disease and in the absence of significant obstructive CAD [224,225,226,227].

## 7. CMD with Obstructive Atherosclerosis

There are close interactions between coronary epicardial and microvascular dysfunction. Structural and functional changes of epicardial and microvascular arterial circulation may be considered expression of the same atherosclerotic process which affects the whole coronary circulation. In most CAD patients, focal and diffuse epicardial and microvascular endothelial dysfunction, expressed by coronary spasm induced by the acetylcholine provocative test, often coexist and may strongly influence each other [29,228].

### 7.1. Epicardial Effects on Microcirculation

Coronary endothelial dysfunction represents an early manifestation of atherosclerosis and may precede the development of obstructive lesions for a long time. Diffuse coronary atherosclerosis without focal stenosis and chronic coronary artery stenosis reduce perfusion pressure along the arterial length, resulting in functional and structural distal microvascular modifications [29,120,229]. These include microvascular remodeling distal to a stenosis and capillary rarefaction, as well as alteration in control of vascular tone which may result in impaired compensatory arteriolar vasodilatation in response to lower perfusion pressure [230,231]. From a clinical perspective, the impairment of microvascular function due to epicardial stenosis may limit the effectiveness of myocardial revascularization and is responsible for the persistence or recurrence of angina in approximately 30% of patients undergoing percutaneous coronary intervention (PCI) [232]. There are conflicting results as to whether coronary revascularization may provide better results than medical therapy alone, even when functional assessment with fractional flow reserve (FFR) is used to guide revascularization instead of angiography visualization [233,234,235]. These observations indicate that epicardial atherosclerosis may induce ischemia symptoms by both increasing proximal artery resistance to blood flow and causing microvascular dysfunction. This may explain the discrepancies between obstructive lesion severity observed on invasive angiography and the extent and severity of myocardial ischemia [6,236].

### 7.2. Coronary No-Reflow

**Figure 3:**
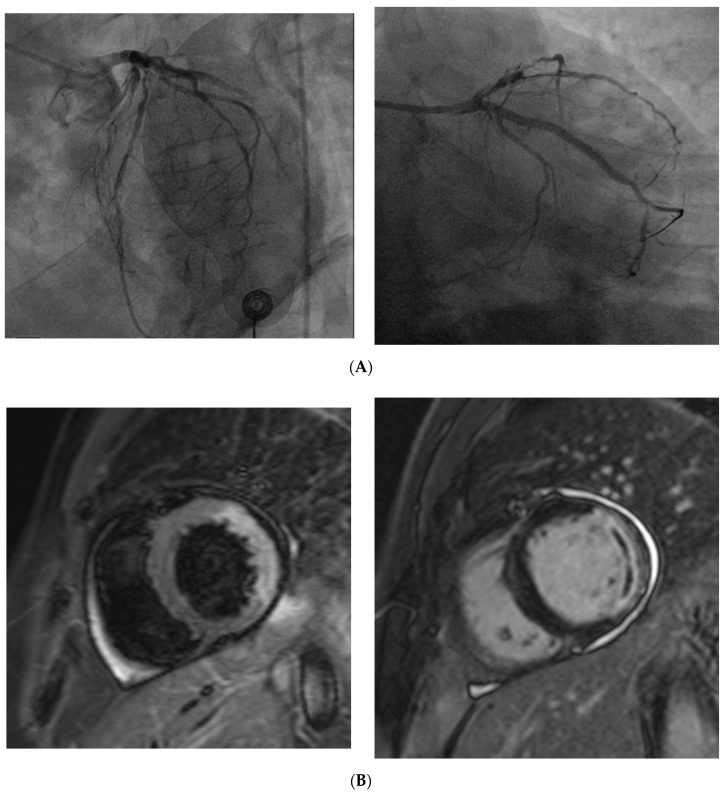
(**A**) Coronary angiography of a 67 year old man presenting with STEMI shows the complete occlusion of a large marginal branch and a severe stenosis in the middle segment of the left anterior descending (left). Coronary angiography of the same patient demonstrates the revascularization of the marginal branch after percutaneous coronary intervention (PTCA) and drug-eluting stent (DES) implantation. (**B**) Cardiac magnetic resonance of the same patient, acquired the day after the PTCA shows alterations in the vascular territory of the previously occluded coronary vessel. Hyperintense signal on T2 turbo inversion recovery magnitude (TIRM) sequence reflects myocardial edema in the anterolateral and inferolateral segments (left). Delayed enhancement (hyperintense signal) indicates transmural infarction in the same myocardial segments (right); the linear hypointense area within the pathological myocardial hyperintensity, referred to as “no-reflow” phenomenon, is consistent with microvascular obstruction. It is due to a persistent perfusion defect despite the epicardial vessel revascularization, and is associated with a worse prognosis.

CMD has an important role in the development of myocardial injury after PCI following ST elevation myocardial infarction (STEMI). About 30–50% of patients undergoing PCI shows reduced or delayed myocardial flow and myocardial perfusion despite epicardial coronary artery recanalization (Figure 3). This condition is termed “no-reflow” microvascular obstruction (MVO), or iatrogenic CMD, and is related to microvascular disorders caused by interruption of blood flow followed by its acute restoration [119,237]. MVO results from a variable combination of vasoconstriction, distal microvascular embolization of atherothrombotic debris originating from epicardial thrombus, ischemic injury with increased endothelial vascular permeability and focal swelling obstructing the lumen, massive infiltration by platelets, inflammatory cells and cholesterol, and vasoconstriction [93,238,239,240]. These microvascular abnormalities reduce or block blood flow and may result in micro-infarcts. Moreover, if microvascular flow is reduced or absent in a necrotic area, necrotic debris will not be removed, and cells and cytokines involved in the healing process will not reach the infarcted area. This may reduce the healing response of the infarcted myocardium, leading to thinning and expansion of the necrotic zone, adverse left ventricular remodeling, and heart failure [166,241,242,243]. Although MVO is associated with poor prognosis, variable degrees of no-reflow occur in almost all patients undergoing PCI and improves spontaneously over time in approximately 50% of patients [244,245].

### 7.3. Microvascular Effects on Epicardial Coronary Arteries

In addition to the effects of epicardial atherosclerosis on microvascular endothelial function, the opposite relationship, in which CMD causes thrombus formation in epicardial coronary arteries, has been proposed [246,247]. CMD may contribute to the development of epicardial atherosclerosis through reduced blood flow, which reduces WSS, leading to progressive endothelial dysfunction of epicardial arteries [248,249]. Endothelial dysfunction of coronary microcirculation and epicardial arteries may represent different stages of coronary atherosclerosis development [250]. At the earliest stage of the disease, conventional CV risk factors may promote coronary microvascular endothelial dysfunction and subclinical atherosclerosis. Later, microvascular dysfunction may induce a reduction in WSS of upstream epicardial arteries, thereby increasing their endothelial inflammation. Afterwards, microvascular endothelial dysfunction and low WSS may result in progression of focal epicardial lesions [243]. Intravascular imaging confirmed the relationship between microvascular and epicardial atherosclerosis, showing that increased microvascular resistance is associated with increased prevalence of atherosclerotic lesions with vulnerable features in the corresponding epicardial arteries [251].

## 8. Diagnosis of CMD

In patients with clinically suspected microvascular angina or vasospastic angina and documented ECG changes, computed tomographic coronary angiography (CTCA) or conventional angiography are indicated to rule out the presence of significant coronary stenosis. Since coronary microcirculation is beyond the resolution of invasive and non-invasive coronary angiography, the diagnosis of CMD is based on functional assessment of the coronary arteries, which can be performed using both invasive and non-invasive methods. The choice of the method depends on the clinical presentation of the patient, acute or chronic, the need for repeated assessments, and the presence of comorbidities.

### 8.1. Invasive Diagnosis

Invasive tests of coronary artery function are the reference standard for the diagnosis of CMD [252]. After excluding significant obstructive CAD angiographically, non-significant epicardial stenoses by measurement of fractional flow reserve (FFR) [253], and epicardial coronary artery spasm as cause for angina, functional disorders of coronary microcirculation are investigated [254].

Patients with chest pain undergoing diagnostic angiography may show delayed flow of contrast, despite the absence of obstructive CAD, called “coronary slow flow phenomenon” or “cardiac syndrome Y” [255,256,257,258]. The slow contrast flow may reflect increased coronary microvascular resistance that contributes to the angina symptoms [259]. Two approaches have been used to define the “slow contrast flow”. The TIMI (thrombolysis in myocardial infarction) flow grade is an index that scores contrast flow from TIMI-0 (no flow) to TIMI-3 (normal flow) [260]. This method is also used in diagnosing the no-reflow phenomenon. An alternative method is the corrected TIMI frame count, based on the number of cine frames required to opacify the distal arterial sites [261].

**Figure 4:**
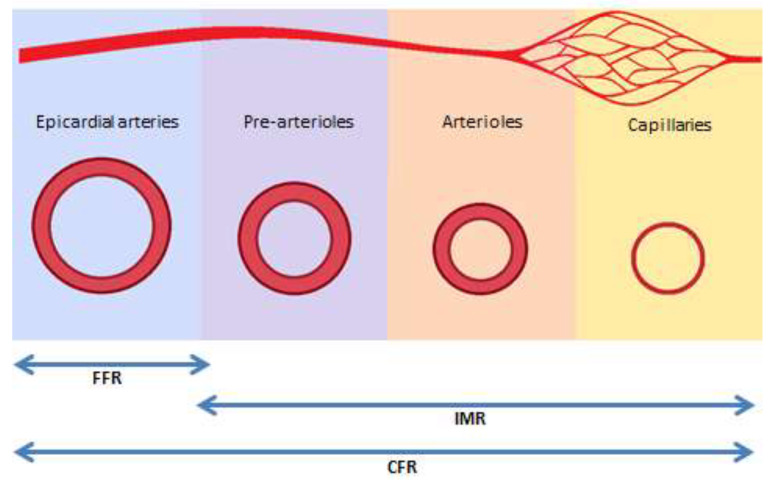
Functional components of invasive diagnostic tests. FFR: fractional flow reserve; IMR: index of microvascular resistance; CFR: coronary flow reserve.

A comprehensive assessment of microvascular function includes testing the two main mechanisms of microvascular dysfunction: (a) impaired endothelium-independent microvascular vasodilatation, which is measured by coronary flow reserve (CFR) and by index of microvascular resistance (IMR), and (b) impaired endothelium-dependent dysfunction, which evaluates the induction of epicardial or microvascular spasm after intracoronary injection of acetylcholine [3,29,254,262,263]. In addition, coronary microcirculation may be also adversely affected by embolization of thrombotic material following percutaneous coronary intervention. Both CFR and IMR are measured using intravenous vasodilators, such as adenosine. In normal conditions, coronary blood flow increases 3–4 times in response to increased myocardial oxygen requirements. CFR is the ability of coronary blood flow to match the metabolic demand and is measured as the ratio of maximal flow after adenosine induced hyperemia to resting absolute myocardial blood flow. CFR reflects the combined vasodilator capacity of epicardial and microvascular coronary arteries. Hence, its interpretation needs assessment of FFR, which estimates the severity of epicardial stenosis (Figure 4). Microvascular resistance, expressed as index of microcirculatory resistance (IMR) may be measured by thermodilution or intravascular Doppler in a hyperemic condition. A more recent method, based on thermodilution and continuous flow of saline (RayFlow catheter), has been developed to calculate the absolute coronary blood flow in a hyperemic condition, and epicardial and microvascular resistance [264]. Compared to IMR and Doppler, continuous thermodilution is not dependent on resting values, is therefore less influenced by hemodynamic changes, and is operator independent.

Although FFR represents the severity of epicardial stenosis and is relatively independent of microvascular function, the presence of significant microvascular disease may increase FFR value for the same level of epicardial stenosis [265]. These patients show discordance between FFR and CFR, which has important clinical relevance [236]. Indeed, in patients with high FFR (>0.80), CFR is determined mainly by the status of the microvascular system. Hence, measurements of CFR and IMR in patients with high FFR are used to assess microvascular function, allowing more accurate risk stratification [266,267]. In these patients, values of CFR < 2.0 or IMR ≥ 25 units indicate an abnormal microvascular function (Table 3) [268]. FFR is measured during maximal hyperemia induced with the intracoronary administration of adenosine, to reduce microvascular resistance, thus using pressure as a surrogate of coronary flow for the assessment of stenosis severity. In recent years, some studies have shown that intracoronary pressure measurement at rest, without the administration of adenosine (instantaneous wave-free ratio or iFR), has diagnostic accuracy similar to that of FFR [269]. As a clinical consequence, among patients with stable angina or acute coronary syndrome, coronary revascularization guided by iFR is non-inferior to revascularization guided by FFR with respect to the risk of major adverse cardiac events [270,271].

Endothelium-dependent dysfunction is assessed using intracoronary acetylcholine infusion. In normal endothelium, acetylcholine induces vasodilatation at both epicardial and microcirculation levels by stimulating NO synthesis. In a dysfunctional endothelium or in impaired smooth muscle cell function, acetylcholine triggers paradoxical arteriolar vasoconstriction [59]. In patients with CMD, acetylcholine infusion may trigger epicardial and/or microvascular spasm with angina symptoms, with or without ECG changes.

### 8.2. Non-Invasive Diagnosis

Non-invasive testing can only evaluate surrogate markers of coronary function. Moreover, contrary to obstructive CAD in which perfusion abnormalities have regional distribution, myocardial impairment in CMD may be a generalized process resulting in diffuse myocardial perfusion abnormalities. Therefore, non-invasive ischemia tests may be normal.

Transthoracic Doppler echocardiography (TTDE) may identify the maximal diastolic flow in the left anterior descending coronary artery at rest and during adenosine/dipyridamole stress, to estimate CFR [272,273,274]. This is the ratio of hyperemic to rest absolute myocardial flow and in patients without epicardial coronary flow limitation CFR is a measure of microvascular function [18]. Despite TTDE being a low-cost, radiation-free, and readily available exam, it lacks accuracy and requires extensive training.

Positron emission tomography (PET) is the reference standard for non-invasive assessment of myocardial blood flow and CFR by quantification of myocardial blood flow (MBF) at rest and during pharmacologically induced maximal hyperemia [275]. In patients without epicardial stenosis, PET may significantly contribute to the assessment of CMD [276]. PET has been largely validated and compared to other invasive and non-invasive tests for ischemic burden assessment in patients with CAD or non-ischemic cardiomyopathies [33,277,278,279]. However, the use of PET is limited by its availability, cost, and exposure to radiation.

Cardiac magnetic resonance (CMR) is also used to assess myocardial perfusion [277]. The high resolution of CMR (1 × 1 mm at 3.0 Tesla) allows for visualization of transmural myocardial flow and assessment of CMD in patients with non-obstructive CAD [280]. The images are acquired at rest and after vasodilator-stress (i.e., adenosine) associated with gadolinium-based contrast agent injection. CMR has shown promising results to distinguish epicardial versus microvascular impairment compared to invasive FFR assessment [281]. Late gadolinium enhancement, detected by CMR, is correlated with higher risk of major CV events and mortality [282,283,284].

In terms of dynamic myocardial perfusion computer tomography (CT), technological advances in cardiac computed tomography allow functional study of the coronary circulation in addition to anatomical data [285,286]. Dynamic CT perfusion acquires multiple CT images as contrast moves through the myocardium, providing results comparable to CMR [277]. After iodinated-contrast media injection, ECG-gated CT scan is performed, allowing estimation of myocardial blood flow [287]. Using this technique could allow functional assessment of both myocardium and the coronary arteries, however at the price of higher radiation exposure.

## 9. Treatment of CMD

Although patients with CMD are at high risk of adverse CV events, particularly in women, practice guideline recommendations for the treatment of these patients are based on weak evidence reflecting the absence of randomized controls [288,289,290]. This is mainly due to the difficulty in accurate diagnosis of CMD and hence the proposal of heterogeneous causes for angina. Control of traditional CV risk factors is the main therapeutic objective to prevent progression of microvascular disease and to reduce the frequency and intensity of ischemic symptoms [100]. Studies with conventional anti-atherosclerosis drugs, including statins, low-dose aspirin, angiotensin-converting enzyme inhibitor (ACE-I), or receptor blockers (ARB), as well as with conventional anti-anginal treatment including calcium antagonists, nitrates, and beta blockers, which are the standard treatment for patients with obstructive CAD, often show conflicting results [100,289]. Most studies included heterogeneous groups of patients, small sample size, different treatments, endpoints, and definition of CMD [291,292]. Overall, anti-anginal drugs are effective in about a half of patients with MVA.

The second line anti-anginal drug ranolazine, which is effective in effort-induced angina in patients refractory to standard anti-ischemic therapy, has been found helpful in MVA patients in three small-sized trials, but ineffective on symptoms or myocardial perfusion in a larger study [293,294]. Women with INOCA with low baseline CFR values may benefit from ACE-I [295]. Ivabradine, a drug that selectively reduces sinus node activity, has been shown to improve angina in MVA patients. However, microvascular function does not change, suggesting that symptomatic improvement is attributable to heart rate lowering effect [296].

In patients with diabetes, sodium–glucose cotransporter-2 (SGLT-2) inhibitors improve endothelial function and reduce major adverse cardiovascular events [297]. However, it is unknown whether this class of medication may improve ischemia and outcomes even in patients with CMD [289].

The effects of anti-inflammatory drugs on CMD have been tested in patients with systemic inflammatory or autoimmune diseases. Although these drugs may block endothelial dysfunction, which has a key role in CMD, the effect of specific treatments are difficult to assess since all anti-ischemic agents currently used for CAD have some degree of anti-inflammatory effect [298]. Interleukins (1β, 18, and 6) are key inflammatory cytokines in the pathogenesis of CAD. Their inhibition by colchicine or receptor antagonist has been shown to improve CFR and myocardial contractility and relaxation in patients with rheumatoid arthritis [299,300]. However it is unknown whether these effects are directly produced by receptor inhibition, and whether they will be maintained over time.

CMD patients are heterogeneous in their clinical presentation, severity, and response to therapies [293]. Understanding this heterogeneity may enable better targeting of treatment to the subgroups of patients most likely to respond. Indeed, a therapeutic strategy of precision (or stratified) medicine based on invasive identification of subgroups of angina patients with distinct mechanisms of disease, linked to personalized treatment, has been implemented with beneficial effects on angina severity and quality of life, compared with standard care [268].

In some patients with CMD and refractory angina despite standard medical therapy, percutaneous implantation of a balloon-expandable device in the coronary sinus creating a pressure gradient, thus favoring the redistribution of blood into ischemic myocardium, resulted in significant improvement of symptoms [301]. Overall, therapies for CMD patients have low levels of evidence. Larger trials and longer follow-up are needed to identify effective treatment.

## 10. Conclusions

A substantial proportion of patients with chest pain undergoing diagnostic coronary angiography do not show significant obstructive coronary lesions. Most of these patients have a combination of functional and structural abnormalities of coronary microcirculation strongly associated with endothelial dysfunction. Despite the absence of significant coronary stenosis, CMD carries increased risk of CV events. There is also evidence that CMD may coexist with obstructive coronary atherosclerosis and may be part of diffuse microvascular disease involving other organs, such as brain and kidney. In addition, impaired coronary microvascular function is strongly associated with HFpEF, diabetes, hypertensive heart disease, hypertrophic cardiomyopathy, aortic stenosis, and chronic inflammatory and autoimmune diseases. Patients with signs and symptoms of myocardial ischemia in the absence of flow-limiting epicardial coronary artery stenosis should undergo invasive coronary pharmacological provocation testing to investigate coronary microvascular function. This may help in identifying specific disease phenotypes within such heterogeneous populations, stratify their CV risk, and evaluate interaction of CMD with CAD. Despite the prognostic relevance of CMD investigation, more studies are needed to define the therapeutic strategies to reduce the development and progression of CMD in these patients.

## Figures and Tables

**Table 1:** Clinical criteria for the diagnosis of microvascular angina (MVA).

1. Symptoms of myocardial ischemia
-Effort and/or rest angina
-Angina equivalents (exertional dyspnea)
2. Absence of obstructive CAD (<50% diameter reduction or FFR > 0.80) by coronary CTA or invasive angiography
3. Objective evidence of myocardial ischemia
-Ischemic ECG changes during chest pain
-Stress-induced chest pain and/or ischemic ECG changes with or without transient
or reversible abnormal myocardial perfusion and/or wall motion abnormality
4. Evidence of impaired coronary microvascular function
-Impaired coronary flow reserve ≤ 2.0
-Coronary microvascular spasm, defined as symptoms and ischemic ECG changes but not epicardial spasm during acetylcholine testing
-Abnormal coronary microvascular resistance indices (IMR > 25)
-Coronary slow flow phenomenon, defined as TIMI frame count > 25

Definitive MVA if all 4 criteria are present. Suspected MVA if symptoms of ischemia with no obstructive coronary artery disease are present (criteria 1 and 2) but only objective evidence of myocardial ischemia (criteria 3) or evidence of impaired coronary microvascular function (criteria 4) alone. CAD = coronary artery disease; ECG = electrocardiogram; CTA computed tomographic angiography; FFR = fractional flow reserve; IMR = index of microcirculatory resistance; TIMI = thrombolysis in myocardial infarction.

**Table 2:** Clinical classifications of coronary microvascular dysfunction (CMD).

A. CMD in chronic coronary syndrome
with non-obstructive chronic coronary syndrome
with obstructive chronic coronary syndrome
CMD in acute coronary syndrome (ACS)
with non-obstructive ACS
with obstructive ACS
with coronary no-reflow phenomenon
CMD following successful revascularization after MI
B. Group 1. CMD in the absence of obstructive CAD and myocardial disease
Group 2. CMD in the presence of myocardial disease
Group 3. CMD in the presence of obstructive CAD
Group 4. CMD after successful percutaneous coronary intervention
C. CMD without atherosclerosis
CMD with non-obstructive atherosclerosis
CMD with obstructive atherosclerosis

**Table 3:** Invasive diagnostic criteria for CMD.

Clinical Feature	Dysfunction	Diagnostic Criteria
Microvascular angina	Impaired coronary vasodilatation	CFR < 2.0
	Increased microvascular resistance	IMR ≥ 25 units
	Microvascular spasm	angina symptoms after intracoronary Ach Ischemic ECG changes <90% diameter reduction
Vasospastic angina	Epicardial spasm	Angina symptoms after intracoronary Ach Infusion and Ischemic ECG changes with >90% epicardial coronary constriction

Exclusion of significant obstructive CAD (FFR > 0.80). FFR: fractional flow reserve; CFR: coronary flow reserve; IMR: index of microvascular resistance; Ach: acetylcholine.
